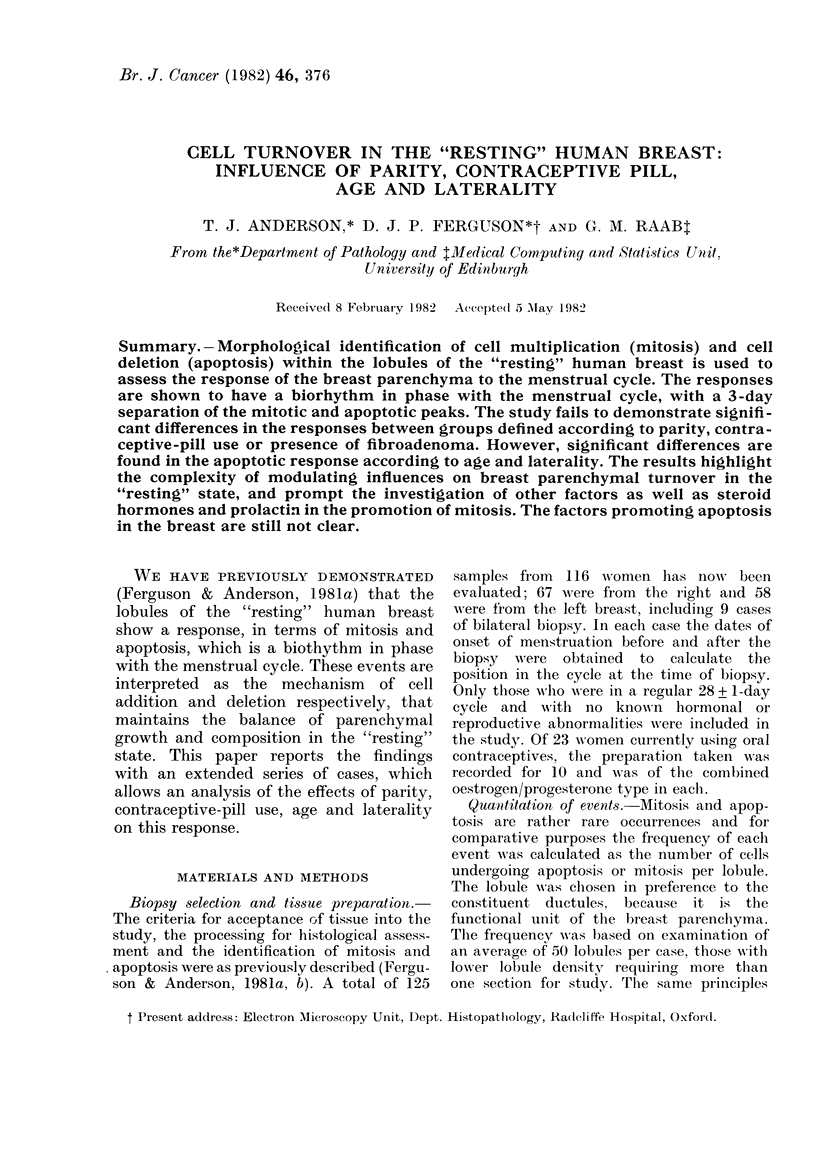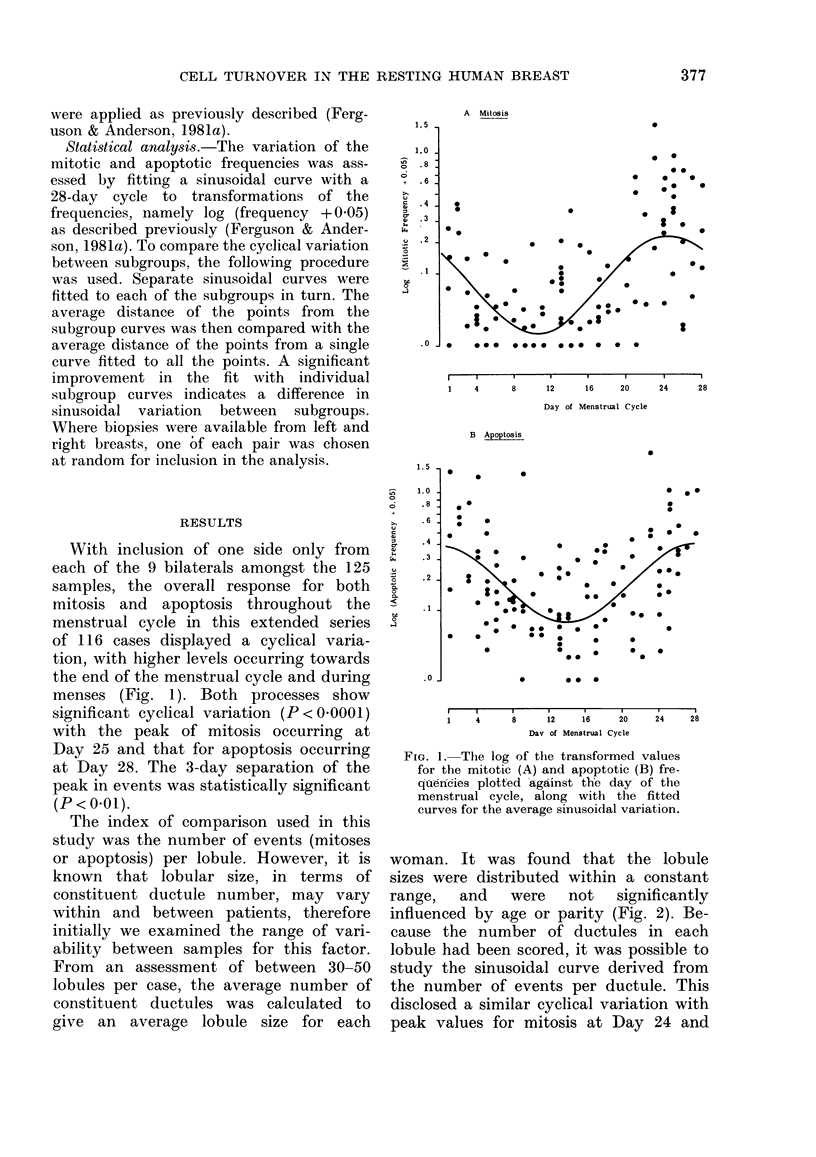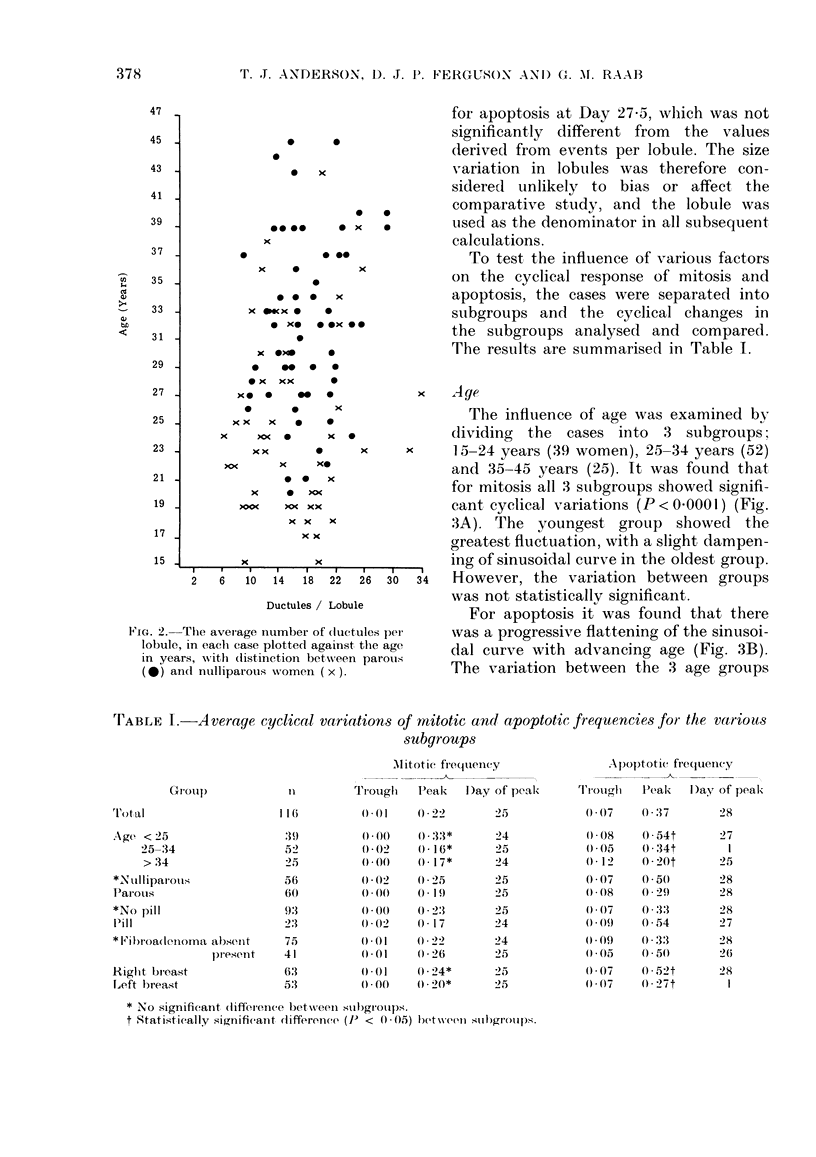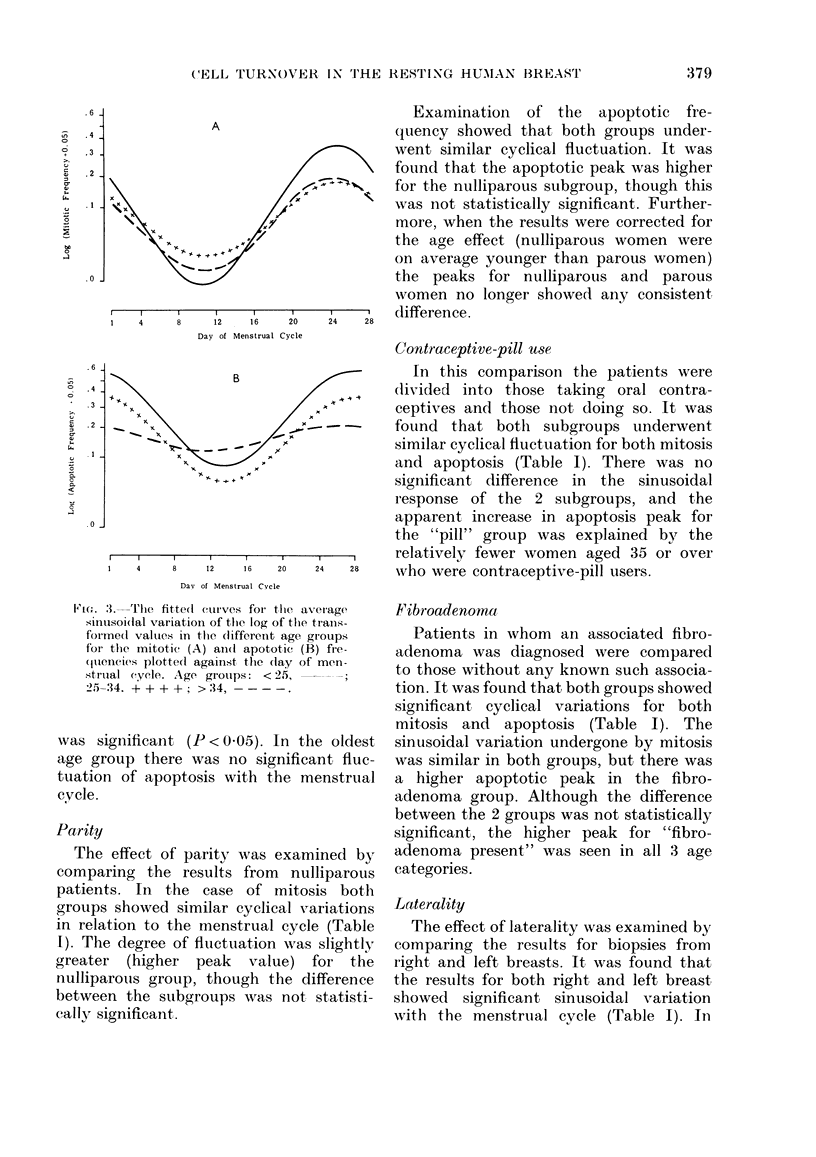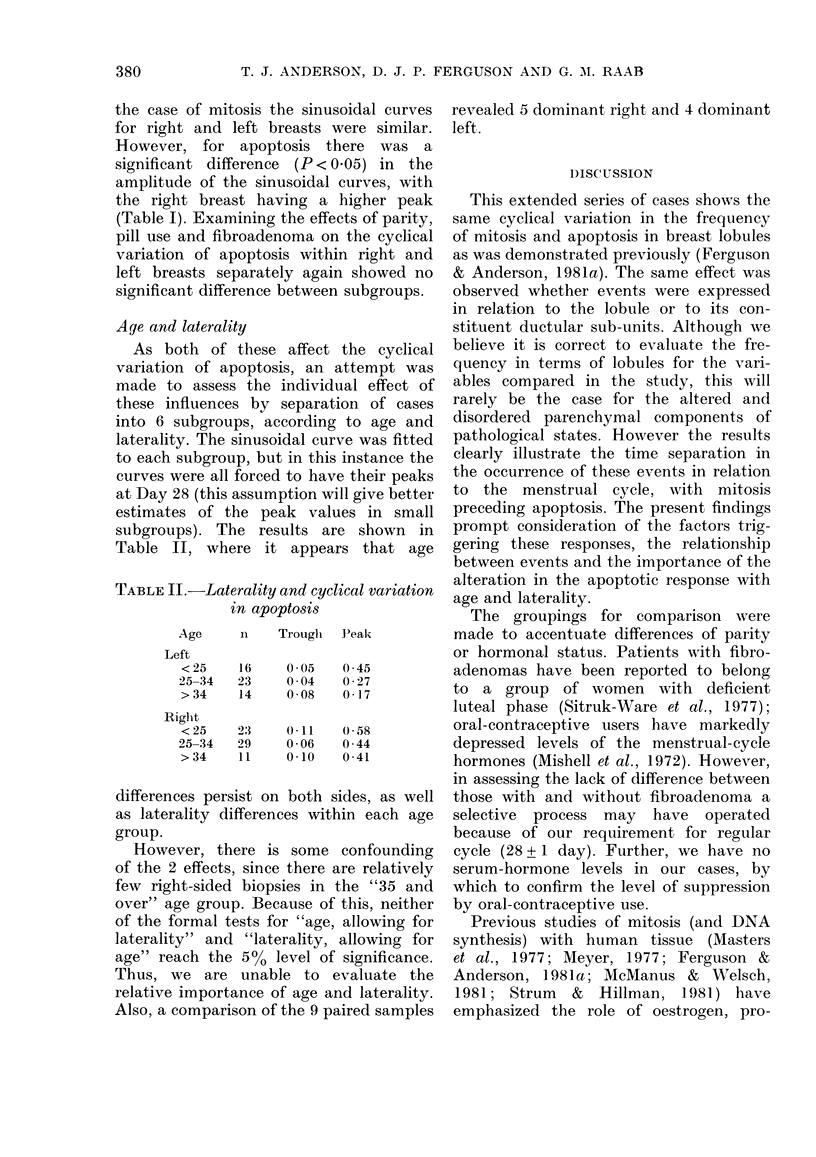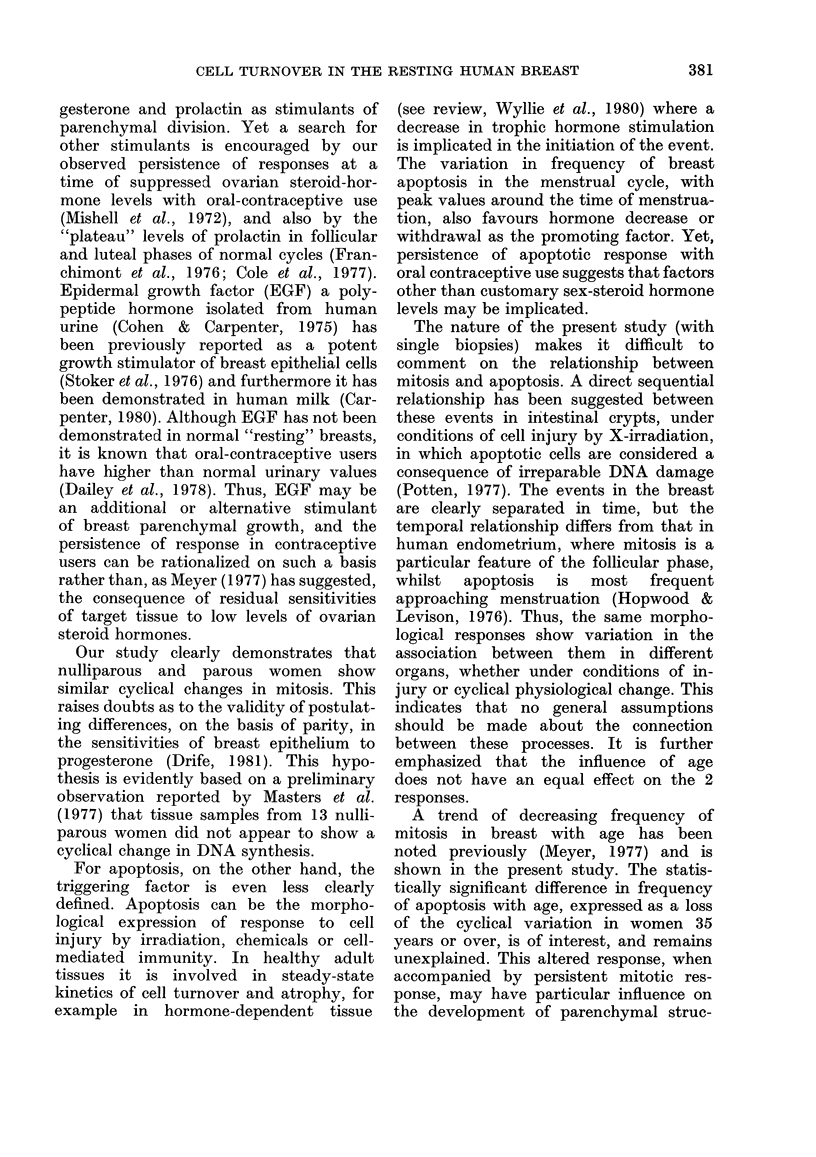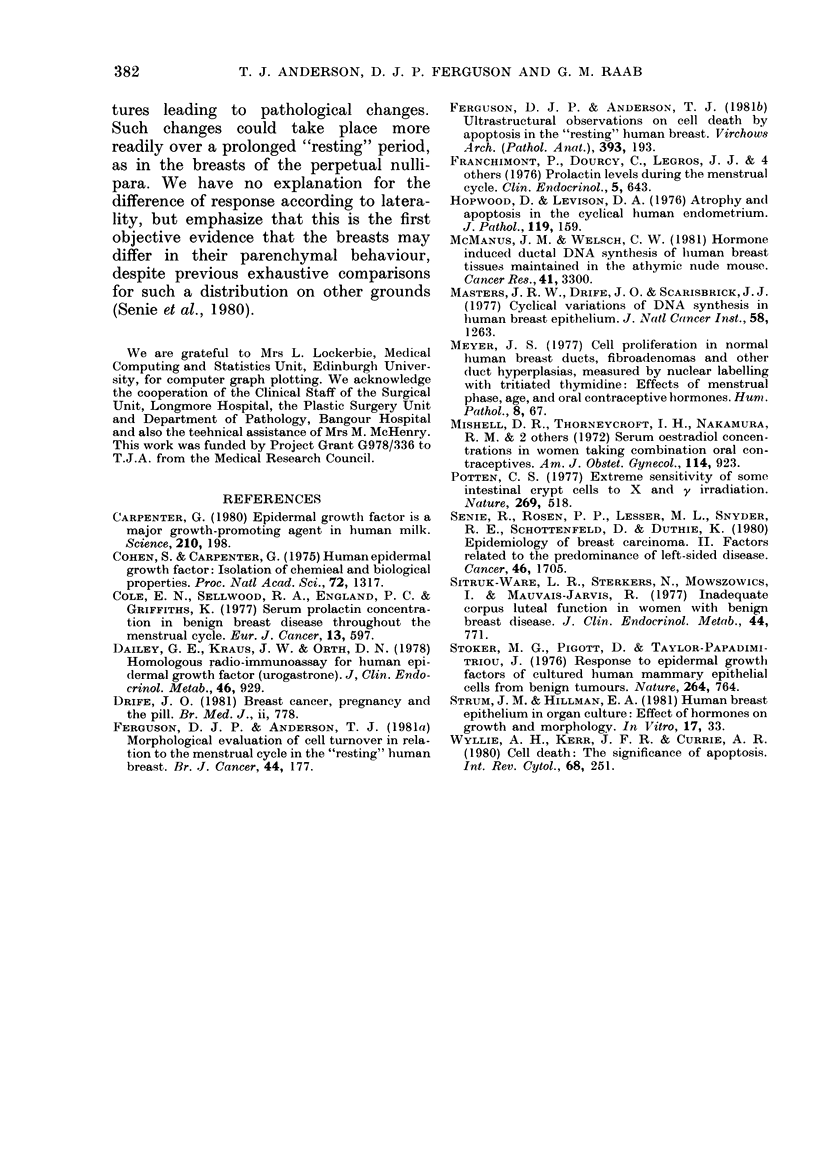# Cell turnover in the "resting" human breast: influence of parity, contraceptive pill, age and laterality.

**DOI:** 10.1038/bjc.1982.213

**Published:** 1982-09

**Authors:** T. J. Anderson, D. J. Ferguson, G. M. Raab

## Abstract

Morphological identification of cell multiplication (mitosis) and cell deletion (apoptosis) within the lobules of the "resting" human breast is used to assess the response of the breast parenchyma to the menstrual cycle. The responses are shown to have a biorhythm in phase with the menstrual cycle, with a 3-day separation of the mitotic and apoptotic peaks. The study fails to demonstrate significant differences in the responses between groups defined according to parity, contraceptive-pill use or presence of fibroadenoma. However, significant differences are found in the apoptotic response according to age and laterality. The results highlight the complexity of modulating influences on breast parenchymal turnover in the "resting" state, and prompt the investigation of other factors as well as steroid hormones and prolactin in the promotion of mitosis. The factors promoting apoptosis in the breast are still not clear.


*Br. J. Cancer* (1982) **46**, 376

### CELL TURNOVER IN THE "RESTING" HUMAN BREAST: INFLUENCE OF PARITY, CONTRACEPTIVE PILL, AGE AND LATERALITY

T. J. ANDERSON,* D. J. P. FERGUSON*† AND G. M. RAAB‡

*From the*Department of Pathology and ‡Medical Computing and Statistics Unit, University of Edinburgh*

Received 8 February 1982 Accepted 5 May 1982

**Summary.—Morphological identification of cell multiplication (mitosis) and cell deletion (apoptosis) within the lobules of the "resting" human breast is used to assess the response of the breast parenchyma to the menstrual cycle. The responses are shown to have a biorhythm in phase with the menstrual cycle, with a 3-day separation of the mitotic and apoptotic peaks. The study fails to demonstrate significant differences in the responses between groups defined according to parity, contraceptive-pill use or presence of fibroadenoma. However, significant differences are found in the apoptotic response according to age and laterality. The results highlight the complexity of modulating influences on breast parenchymal turnover in the "resting" state, and prompt the investigation of other factors as well as steroid hormones and prolactin in the promotion of mitosis. The factors promoting apoptosis in the breast are still not clear.**

WE HAVE PREVIOUSLY DEMONSTRATED (Ferguson & Anderson, 1981*a*) that the lobules of the "resting" human breast show a response, in terms of mitosis and apoptosis, which is a biothythm in phase with the menstrual cycle. These events are interpreted as the mechanism of cell addition and deletion respectively, that maintains the balance of parenchymal growth and composition in the "resting" state. This paper reports the findings with an extended series of cases, which allows an analysis of the effects of parity, contraceptive-pill use, age and laterality on this response.

### MATERIALS AND METHODS

*Biopsy selection and tissue preparation.*—The criteria for acceptance of tissue into the study, the processing for histological assessment and the identification of mitosis and apoptosis were as previously described (Ferguson & Anderson, 1981*a*, *b*). A total of 125 samples from 116 women has now been evaluated; 67 were from the right and 58 were from the left breast, including 9 cases of bilateral biopsy. In each case the dates of onset of menstruation before and after the biopsy were obtained to calculate the position in the cycle at the time of biopsy. Only those who were in a regular 28 ± 1-day cycle and with no known hormonal or reproductive abnormalities were included in the study. Of 23 women currently using oral contraceptives, the preparation taken was recorded for 10 and was of the combined oestrogen/progesterone type in each.

*Quantitation of events.*—Mitosis and apoptosis are rather rare occurrences and for comparative purposes the frequency of each event was calculated as the number of cells undergoing apoptosis or mitosis per lobule. The lobule was chosen in preference to the constituent ductules, because it is the functional unit of the breast parenchyma. The frequency was based on examination of an average of 50 lobules per case, those with lower lobule density requiring more than one section for study. The same principles

† Present address: Electron Microscopy Unit, Dept. Histopathology, Radcliffe Hospital, Oxford.

were applied as previously described (Ferguson & Anderson, 1981*a*).

*Statistical analysis.*—The variation of the mitotic and apoptotic frequencies was assessed by fitting a sinusoidal curve with a 28-day cycle to transformations of the frequencies, namely log (frequency $+0{\cdot}05$) as described previously (Ferguson & Anderson, 1981*a*). To compare the cyclical variation between subgroups, the following procedure was used. Separate sinusoidal curves were fitted to each of the subgroups in turn. The average distance of the points from the subgroup curves was then compared with the average distance of the points from a single curve fitted to all the points. A significant improvement in the fit with individual subgroup curves indicates a difference in sinusoidal variation between subgroups. Where biopsies were available from left and right breasts, one of each pair was chosen at random for inclusion in the analysis.

Fig. 1.—The log of the transformed values for the mitotic (A) and apoptotic (B) frequencies plotted against the day of the menstrual cycle, along with the fitted curves for the average sinusoidal variation.

### RESULTS

With inclusion of one side only from each of the 9 bilaterals amongst the 125 samples, the overall response for both mitosis and apoptosis throughout the menstrual cycle in this extended series of 116 cases displayed a cyclical variation, with higher levels occurring towards the end of the menstrual cycle and during menses (Fig. 1). Both processes show significant cyclical variation ($P<0{\cdot}0001$) with the peak of mitosis occurring at Day 25 and that for apoptosis occurring at Day 28. The 3-day separation of the peak in events was statistically significant ($P<0{\cdot}01$).

The index of comparison used in this study was the number of events (mitoses or apoptosis) per lobule. However, it is known that lobular size, in terms of constituent ductule number, may vary within and between patients, therefore initially we examined the range of variability between samples for this factor. From an assessment of between 30–50 lobules per case, the average number of constituent ductules was calculated to give an average lobule size for each woman. It was found that the lobule sizes were distributed within a constant range, and were not significantly influenced by age or parity (Fig. 2). Because the number of ductules in each lobule had been scored, it was possible to study the sinusoidal curve derived from the number of events per ductule. This disclosed a similar cyclical variation with peak values for mitosis at Day 24 and

FIG. 2.—The average number of ductules per lobule, in each case plotted against the age in years, with distinction between parous (●) and nulliparous women (×).

for apoptosis at Day 27·5, which was not significantly different from the values derived from events per lobule. The size variation in lobules was therefore considered unlikely to bias or affect the comparative study, and the lobule was used as the denominator in all subsequent calculations.

To test the influence of various factors on the cyclical response of mitosis and apoptosis, the cases were separated into subgroups and the cyclical changes in the subgroups analysed and compared. The results are summarised in Table I.

*Age*

The influence of age was examined by dividing the cases into 3 subgroups; 15–24 years (39 women), 25–34 years (52) and 35–45 years (25). It was found that for mitosis all 3 subgroups showed significant cyclical variations ($P < 0{\cdot}0001$) (Fig. 3A). The youngest group showed the greatest fluctuation, with a slight dampening of sinusoidal curve in the oldest group. However, the variation between groups was not statistically significant.

For apoptosis it was found that there was a progressive flattening of the sinusoidal curve with advancing age (Fig. 3B). The variation between the 3 age groups

TABLE I.—*Average cyclical variations of mitotic and apoptotic frequencies for the various subgroups*

| Group | n | Mitotic frequency Trough | Peak | Day of peak | Apoptotic frequency Trough | Peak | Day of peak |
|---|---|---|---|---|---|---|---|
| Total | 116 | 0·01 | 0·22 | 25 | 0·07 | 0·37 | 28 |
| Age < 25 | 39 | 0·00 | 0·33* | 24 | 0·08 | 0·54† | 27 |
| 25–34 | 52 | 0·02 | 0·16* | 25 | 0·05 | 0·34† | 1 |
| > 34 | 25 | 0·00 | 0·17* | 24 | 0·12 | 0·20† | 25 |
| *Nulliparous | 56 | 0·02 | 0·25 | 25 | 0·07 | 0·50 | 28 |
| Parous | 60 | 0·00 | 0·19 | 25 | 0·08 | 0·29 | 28 |
| *No pill | 93 | 0·00 | 0·23 | 25 | 0·07 | 0·33 | 28 |
| Pill | 23 | 0·02 | 0·17 | 24 | 0·09 | 0·54 | 27 |
| *Fibroadenoma absent | 75 | 0·01 | 0·22 | 24 | 0·09 | 0·33 | 28 |
| present | 41 | 0·01 | 0·26 | 25 | 0·05 | 0·50 | 26 |
| Right breast | 63 | 0·01 | 0·24* | 25 | 0·07 | 0·52† | 28 |
| Left breast | 53 | 0·00 | 0·20* | 25 | 0·07 | 0·27† | 1 |

* No significant difference between subgroups.
† Statistically significant difference ($P < 0{\cdot}05$) between subgroups.

FIG. 3.—The fitted curves for the average sinusoidal variation of the log of the transformed values in the different age groups for the mitotic (A) and apototic (B) frequencies plotted against the day of menstrual cycle. Age groups: <25, ———; 25–34, + + + +; >34, – – – –.

was significant ($P < 0{\cdot}05$). In the oldest age group there was no significant fluctuation of apoptosis with the menstrual cycle.

*Parity*

The effect of parity was examined by comparing the results from nulliparous patients. In the case of mitosis both groups showed similar cyclical variations in relation to the menstrual cycle (Table I). The degree of fluctuation was slightly greater (higher peak value) for the nulliparous group, though the difference between the subgroups was not statistically significant.

Examination of the apoptotic frequency showed that both groups underwent similar cyclical fluctuation. It was found that the apoptotic peak was higher for the nulliparous subgroup, though this was not statistically significant. Furthermore, when the results were corrected for the age effect (nulliparous women were on average younger than parous women) the peaks for nulliparous and parous women no longer showed any consistent difference.

*Contraceptive-pill use*

In this comparison the patients were divided into those taking oral contraceptives and those not doing so. It was found that both subgroups underwent similar cyclical fluctuation for both mitosis and apoptosis (Table I). There was no significant difference in the sinusoidal response of the 2 subgroups, and the apparent increase in apoptosis peak for the "pill" group was explained by the relatively fewer women aged 35 or over who were contraceptive-pill users.

*Fibroadenoma*

Patients in whom an associated fibroadenoma was diagnosed were compared to those without any known such association. It was found that both groups showed significant cyclical variations for both mitosis and apoptosis (Table I). The sinusoidal variation undergone by mitosis was similar in both groups, but there was a higher apoptotic peak in the fibroadenoma group. Although the difference between the 2 groups was not statistically significant, the higher peak for "fibroadenoma present" was seen in all 3 age categories.

*Laterality*

The effect of laterality was examined by comparing the results for biopsies from right and left breasts. It was found that the results for both right and left breast showed significant sinusoidal variation with the menstrual cycle (Table I). In the case of mitosis the sinusoidal curves for right and left breasts were similar. However, for apoptosis there was a significant difference ($P < 0{\cdot}05$) in the amplitude of the sinusoidal curves, with the right breast having a higher peak (Table I). Examining the effects of parity, pill use and fibroadenoma on the cyclical variation of apoptosis within right and left breasts separately again showed no significant difference between subgroups.

*Age and laterality*

As both of these affect the cyclical variation of apoptosis, an attempt was made to assess the individual effect of these influences by separation of cases into 6 subgroups, according to age and laterality. The sinusoidal curve was fitted to each subgroup, but in this instance the curves were all forced to have their peaks at Day 28 (this assumption will give better estimates of the peak values in small subgroups). The results are shown in Table II, where it appears that age

TABLE II.—*Laterality and cyclical variation in apoptosis*

| Age | n | Trough | Peak |
|---|---|---|---|
| Left | | | |
| <25 | 16 | 0·05 | 0·45 |
| 25–34 | 23 | 0·04 | 0·27 |
| >34 | 14 | 0·08 | 0·17 |
| Right | | | |
| <25 | 23 | 0·11 | 0·58 |
| 25–34 | 29 | 0·06 | 0·44 |
| >34 | 11 | 0·10 | 0·41 |

differences persist on both sides, as well as laterality differences within each age group.

However, there is some confounding of the 2 effects, since there are relatively few right-sided biopsies in the "35 and over" age group. Because of this, neither of the formal tests for "age, allowing for laterality" and "laterality, allowing for age" reach the 5% level of significance. Thus, we are unable to evaluate the relative importance of age and laterality. Also, a comparison of the 9 paired samples revealed 5 dominant right and 4 dominant left.

### DISCUSSION

This extended series of cases shows the same cyclical variation in the frequency of mitosis and apoptosis in breast lobules as was demonstrated previously (Ferguson & Anderson, 1981*a*). The same effect was observed whether events were expressed in relation to the lobule or to its constituent ductular sub-units. Although we believe it is correct to evaluate the frequency in terms of lobules for the variables compared in the study, this will rarely be the case for the altered and disordered parenchymal components of pathological states. However the results clearly illustrate the time separation in the occurrence of these events in relation to the menstrual cycle, with mitosis preceding apoptosis. The present findings prompt consideration of the factors triggering these responses, the relationship between events and the importance of the alteration in the apoptotic response with age and laterality.

The groupings for comparison were made to accentuate differences of parity or hormonal status. Patients with fibroadenomas have been reported to belong to a group of women with deficient luteal phase (Sitruk-Ware *et al.*, 1977); oral-contraceptive users have markedly depressed levels of the menstrual-cycle hormones (Mishell *et al.*, 1972). However, in assessing the lack of difference between those with and without fibroadenoma a selective process may have operated because of our requirement for regular cycle ($28 \pm 1$ day). Further, we have no serum-hormone levels in our cases, by which to confirm the level of suppression by oral-contraceptive use.

Previous studies of mitosis (and DNA synthesis) with human tissue (Masters *et al.*, 1977; Meyer, 1977; Ferguson & Anderson, 1981*a*; McManus & Welsch, 1981; Strum & Hillman, 1981) have emphasized the role of oestrogen, progesterone and prolactin as stimulants of parenchymal division. Yet a search for other stimulants is encouraged by our observed persistence of responses at a time of suppressed ovarian steroid-hormone levels with oral-contraceptive use (Mishell *et al.*, 1972), and also by the "plateau" levels of prolactin in follicular and luteal phases of normal cycles (Franchimont *et al.*, 1976; Cole *et al.*, 1977). Epidermal growth factor (EGF) a polypeptide hormone isolated from human urine (Cohen & Carpenter, 1975) has been previously reported as a potent growth stimulator of breast epithelial cells (Stoker *et al.*, 1976) and furthermore it has been demonstrated in human milk (Carpenter, 1980). Although EGF has not been demonstrated in normal "resting" breasts, it is known that oral-contraceptive users have higher than normal urinary values (Dailey *et al.*, 1978). Thus, EGF may be an additional or alternative stimulant of breast parenchymal growth, and the persistence of response in contraceptive users can be rationalized on such a basis rather than, as Meyer (1977) has suggested, the consequence of residual sensitivities of target tissue to low levels of ovarian steroid hormones.

Our study clearly demonstrates that nulliparous and parous women show similar cyclical changes in mitosis. This raises doubts as to the validity of postulating differences, on the basis of parity, in the sensitivities of breast epithelium to progesterone (Drife, 1981). This hypothesis is evidently based on a preliminary observation reported by Masters *et al.* (1977) that tissue samples from 13 nulliparous women did not appear to show a cyclical change in DNA synthesis.

For apoptosis, on the other hand, the triggering factor is even less clearly defined. Apoptosis can be the morphological expression of response to cell injury by irradiation, chemicals or cell-mediated immunity. In healthy adult tissues it is involved in steady-state kinetics of cell turnover and atrophy, for example in hormone-dependent tissue (see review, Wyllie *et al.*, 1980) where a decrease in trophic hormone stimulation is implicated in the initiation of the event. The variation in frequency of breast apoptosis in the menstrual cycle, with peak values around the time of menstruation, also favours hormone decrease or withdrawal as the promoting factor. Yet, persistence of apoptotic response with oral contraceptive use suggests that factors other than customary sex-steroid hormone levels may be implicated.

The nature of the present study (with single biopsies) makes it difficult to comment on the relationship between mitosis and apoptosis. A direct sequential relationship has been suggested between these events in intestinal crypts, under conditions of cell injury by X-irradiation, in which apoptotic cells are considered a consequence of irreparable DNA damage (Potten, 1977). The events in the breast are clearly separated in time, but the temporal relationship differs from that in human endometrium, where mitosis is a particular feature of the follicular phase, whilst apoptosis is most frequent approaching menstruation (Hopwood & Levison, 1976). Thus, the same morphological responses show variation in the association between them in different organs, whether under conditions of injury or cyclical physiological change. This indicates that no general assumptions should be made about the connection between these processes. It is further emphasized that the influence of age does not have an equal effect on the 2 responses.

A trend of decreasing frequency of mitosis in breast with age has been noted previously (Meyer, 1977) and is shown in the present study. The statistically significant difference in frequency of apoptosis with age, expressed as a loss of the cyclical variation in women 35 years or over, is of interest, and remains unexplained. This altered response, when accompanied by persistent mitotic response, may have particular influence on the development of parenchymal structures leading to pathological changes. Such changes could take place more readily over a prolonged "resting" period, as in the breasts of the perpetual nullipara. We have no explanation for the difference of response according to laterality, but emphasize that this is the first objective evidence that the breasts may differ in their parenchymal behaviour, despite previous exhaustive comparisons for such a distribution on other grounds (Senie *et al.*, 1980).

We are grateful to Mrs L. Lockerbie, Medical Computing and Statistics Unit, Edinburgh University, for computer graph plotting. We acknowledge the cooperation of the Clinical Staff of the Surgical Unit, Longmore Hospital, the Plastic Surgery Unit and Department of Pathology, Bangour Hospital and also the teehnical assistance of Mrs M. McHenry. This work was funded by Project Grant G978/336 to T.J.A. from the Medical Research Council.

### REFERENCES

CARPENTER, G. (1980) Epidermal growth factor is a major growth-promoting agent in human milk. *Science*, **210**, 198.

COHEN, S. & CARPENTER, G. (1975) Human epidermal growth factor: Isolation of chemieal and biological properties. *Proc. Natl Acad. Sci.*, **72**, 1317.

COLE, E. N., SELLWOOD, R. A., ENGLAND, P. C. & GRIFFITHS, K. (1977) Serum prolactin concentration in benign breast disease throughout the menstrual cycle. *Eur. J. Cancer*, **13**, 597.

DAILEY, G. E., KRAUS, J. W. & ORTH, D. N. (1978) Homologous radio-immunoassay for human epidermal growth factor (urogastrone). *J, Clin. Endocrinol. Metab.*, **46**, 929.

DRIFE, J. O. (1981) Breast cancer, pregnancy and the pill. *Br. Med. J.*, ii, 778.

FERGUSON, D. J. P. & ANDERSON, T. J. (1981*a*) Morphological evaluation of cell turnover in relation to the menstrual cycle in the "resting" human breast. *Br. J. Cancer*, **44**, 177.

FERGUSON, D. J. P. & ANDERSON, T. J. (1981*b*) Ultrastructural observations on cell death by apoptosis in the "resting" human breast. *Virchows Arch.* (*Pathol. Anat.*), **393**, 193.

FRANCHIMONT, P., DOURCY, C., LEGROS, J. J. & 4 others (1976) Prolactin levels during the menstrual cycle. *Clin. Endocrinol.*, **5**, 643.

HOPWOOD, D. & LEVISON, D. A. (1976) Atrophy and apoptosis in the cyclical human endometrium. *J. Pathol.*, **119**, 159.

MCMANUS, J. M. & WELSCH, C. W. (1981) Hormone induced ductal DNA synthesis of human breast tissues maintained in the athymic nude mouse. *Cancer Res.*, **41**, 3300.

MASTERS, J. R. W., DRIFE, J. O. & SCARISBRICK, J. J. (1977) Cyclical variations of DNA synthesis in human breast epithelium. *J. Natl Cancer Inst.*, **58**, 1263.

MEYER, J. S. (1977) Cell proliferation in normal human breast ducts, fibroadenomas and other duct hyperplasias, measured by nuclear labelling with tritiated thymidine: Effects of menstrual phase, age, and oral contraceptive hormones. *Hum. Pathol.*, **8**, 67.

MISHELL, D. R., THORNEYCROFT, I. H., NAKAMURA, R. M. & 2 others (1972) Serum oestradiol concentrations in women taking combination oral contraceptives. *Am. J. Obstet. Gynecol.*, **114**, 923.

POTTEN, C. S. (1977) Extreme sensitivity of some intestinal crypt cells to X and $\gamma$ irradiation. *Nature*, **269**, 518.

SENIE, R., ROSEN, P. P., LESSER, M. L., SNYDER, R. E., SCHOTTENFELD, D. & DUTHIE, K. (1980) Epidemiology of breast carcinoma. II. Factors related to the predominance of left-sided disease. *Cancer*, **46**, 1705.

SITRUK-WARE, L. R., STERKERS, N., MOWSZOWICS, I. & MAUVAIS-JARVIS, R. (1977) Inadequate corpus luteal function in women with benign breast disease. *J. Clin. Endocrinol. Metab.*, **44**, 771.

STOKER, M. G., PIGOTT, D. & TAYLOR-PAPADIMITRIOU, J. (1976) Response to epidermal growth factors of cultured human mammary epithelial cells from benign tumours. *Nature*, **264**, 764.

STRUM, J. M. & HILLMAN, E. A. (1981) Human breast epithelium in organ culture: Effect of hormones on growth and morphology. *In Vitro*, **17**, 33.

WYLLIE, A. H., KERR, J. F. R. & CURRIE, A. R. (1980) Cell death: The significance of apoptosis. *Int. Rev. Cytol.*, **68**, 251.